# Study on Fatigue Crack Growth in Rail Steel at Numerical and Experimental Approaches

**DOI:** 10.3390/ma16082981

**Published:** 2023-04-09

**Authors:** Bing Yang, Shuancheng Wang, Jian Li, Xianwang Ding, Qian Xiao, Shoune Xiao

**Affiliations:** 1 State Key Laboratory of Performance Monitoring and Protecting of Rail Transit Infrastructure, East China Jiaotong University, Nanchang 330013, China; 2 State Key Laboratory of Traction Power, Southwest Jiaotong University, Chengdu 610031, China

**Keywords:** rail steel, crack tip plastic zone, CJP model, crack closure, crack propagation rate

## Abstract

Affected by the service environment, the actual service conditions of rail steel are complex, and the safety evaluation methods are limited. In this study, the fatigue crack propagation in the U71MnG rail steel crack tip was analysed by means of the DIC method, focusing on the shielding effect of the plastic zone at the crack tip. The crack propagation in the steel was analysed based on a microstructural approach. The results show that the maximum value of stress of the wheel–rail static contact and rolling contact is in the subsurface of the rail. The test grain size of the material selected along the L–T direction is smaller than that in the L–S one. Within a unit distance, if the grain size is smaller, the number of grains or grain boundaries will be greater so that the driving force required for a crack to pass through the grain boundary barriers will be larger. The Christopher–James–Patterson (CJP) model can well describe the contour of the plastic zone and can well characterize the influence of crack tip compatible stress and the crack closure effect on crack propagation under different stress ratios. The crack growth rate curve at the high-stress ratio is shifted to the left relative to the low-stress ratio, and the crack growth rate curves obtained under different sampling methods have good normalization.

## 1. Introduction

With the development of railways and the progress of metallurgical technology, the structure of rail transit lines faces new challenges in high speed, heavy load, safety and longevity. As the main component of railway track, the U71MnG rail is used to guide wheels, bear train load and provide a continuous, smooth and minimum resistance rolling surface for wheels. In electrified railways or automatic block sections, rails can also be used as track circuits. Affected by the complex service environment, the fracture failure behavior of rail is complex, and the existing safety evaluation methods are limited and susceptible to subjective factors, which may lead to inaccurate evaluation results and threaten operational safety [1].

For the U71MnG rail material in service, there is a certain range in the plastic deformation zone at the crack tip due to stress concentration after external load. Accurately characterizing the shape, location and size of the plastic zone at the crack tip can provide an accurate stress field for the solution of linear elastic fracture mechanics and provide mechanical analysis for accurately evaluating the crack instability and propagation of rail steel materials. The traditional linear elastic fracture mechanics uses the stress intensity factor (SIF) “*K*” to characterize the driving force of the crack tip. The stress field at the crack tip is determined by *K*; the *K* value is proportional to the strength of the crack tip stress field [2]. Affected by dislocation, passivation, hardening, crack bifurcation and other factors, there are many models to characterize the stress field at the crack tip, such as the Westergaard model, the Williams model, the Muskhelishvili model, the Irwin model, the Dugdale model, the Elber model, the Christopher–James–Patterson (CJP) model, etc. [3,4,5,6,7], which have their advantages in characterizing the plastic zone at the crack tip, and the appropriate model can quantify and separate the various factors affecting fatigue crack growth (FCG). As a measurement technique that can intuitively characterize the crack stress field, the photoelastic method can more directly evaluate the influence of crack closure and wake area on the crack tip stress field and SIF. This method can be used to have a deeper understanding of the relationship between wake contact force, effective SIF and specimen flexibility. Based on the Muskhelishvili potential function method, Pacey et al. [8] established a mathematical model considering the uniform compressive stress between the crack surfaces and the shear stress on the crack side. The uniform compressive stress is not an accurate reflection of the contact stress of the crack surface, but it can reflect the influence of the plastic zone on the elastic zone. The variables included in the optimization model make the theoretical stress field agree well with the experimental stress field so that the theoretical model can reflect the influence of the plastic zone and wake zone on the SIF. The model is first applied to the measurement of the SIF of the artificial sharp notch, and the results show that the obtained SIF is in good agreement with the theoretical SIF [9]. Vasco-Olm et al. [10] used 2024-T3 aluminum alloy CT specimens and collected the displacement field of the loading stage under a certain cycle at the DIC method. The Westergaard model, the Williams model, the Muskhelishvili model and the CJP model were used to fit the experimental displacement field to obtain the SIF of each model. The results show that the SIF of the Westergaard model is the largest difference from the theoretical solution. The SIF of the Williams model and the Muskhelishvili model are close to the theoretical solution, while the CJP model is considered to have the ability to evaluate the crack shielding effect. The fracture toughness can be estimated from the variation of *K*_F_ and *K*_R_ with the external load.

In this paper, the fatigue crack propagation in the U71MnG rail steel is studied. The local stress and strain state of rail steel is obtained by the finite element method, and the local service equivalent load environment and boundary condition spectrum are constructed. The DIC method is used as a test method to solve the crack tip displacement field based on the CJP model to obtain the SIF of each stage of crack propagation, accurately characterize the crack propagation behavior of rail steel, and focus on the shielding effect of the plastic zone at the crack tip, combined with the microstructure analysis results to explain the crack propagation behavior of rail steel.

## 2. Wheel–Rail Model Establishment

**Figure 1:**
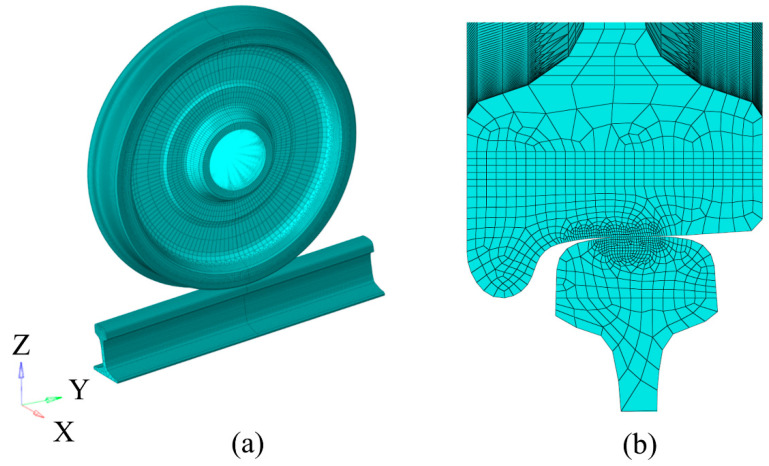
Three-dimensional wheel-rail rolling contact finite element model. (**a**) wheel-rail rolling contact overall model, (**b**) local refinement of contact area.

To determine the location of the experimental sampling and the rationality of the sampling, the finite element method was used to establish a three-dimensional wheel–rail rolling contact. The difference between the rail in the static and rolling state of the wheel was also analysed. The model used a 60 kg/m rail and a LM wear-type tread wheel, the three-dimensional wheel–rail rolling contact finite element model is shown in Figure 1. The X-axis is the rail transverse, the Y-axis is the rail longitudinal, and the Z-axis is the rail vertical coordinate system. The wheel tread and rail profile are both actual sizes, which are divided by full hexahedral mesh. The nominal rolling circle of the wheel is in contact with the center line of the rail. The radius of the wheel is 420 mm, the length of the rail is 500 mm, and the center position of the wheel is 50 mm away from the left end of the rail, which eliminates the influence of the rail boundary conditions on the stress distribution.

The calculation area of wheel–rail contact is a highly nonlinear problem, and it requires high calculation accuracy. To reduce the sensitivity of the contact area to the mesh size, only the mesh of the wheel–rail contact area is refined [11], and the minimum size is 1 mm × 1 mm. The finite element calculation of wheel–rail rolling contact is divided into two stages by using a transient solver. The first stage is the initial contact stage of the wheel–rail, and the second stage is the rolling contact stage of the wheel–rail. In the first stage, there is no relative sliding of the wheel. Under loading, the wheel and rail establish static tight contact. The calculation results of the first stage are used as the initial conditions of the second stage to simulate the wheel rolling along the rail in the positive direction of Y [12]. The node of the hub hole adopts the cp element to couple the Y direction, the Z direction and the rotation freedom around the X axis with the wheel center node. The axle load is 20 t, each wheel is subjected to 100 kN and this loading is added to the wheel center node. In the whole process of calculation, the left side of the rail constrains the degree of freedom in the X and Y directions, the right side constrains the degree of freedom in the X direction and the full constraint at the bottom. In the first stage, the wheel only releases the degree of freedom in the Z direction. In the second stage, the degree of freedom in the Y direction and the angle of constraint rotation around the X axis are released.

**Figure 2:**
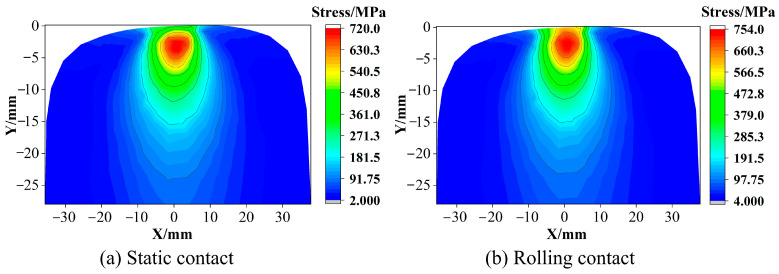
Stress distribution of rail cross section.

**Figure 3:**
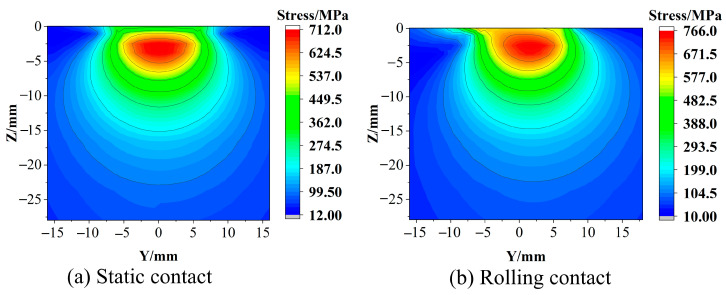
Rail longitudinal section stress distribution.

The wheel–rail contact center position is set as the origin, and the rail stress distribution results of the wheel–rail under static contact and rolling contact are obtained. The distribution of the stress field in the cross section of the two contact states is symmetrical, as shown in Figure 2. However, in the distribution of the longitudinal section, the static state is symmetrical, and the rolling state is asymmetrical, as shown in Figure 3. When the wheel–rail is in static contact, the state near the wheel–rail contact center is mainly adhesive [13]. The area far away from the contact center will have relative displacement due to the deformation of the rail. The area of these areas is small, and the contact pressure is relatively small, resulting in the size and active area of the friction force being small, so the influence on the stress distribution is small, and the stress distribution of the rail is symmetrical. In the rolling contact, the wheel–rail mainly produces relative motion in the longitudinal direction, and the relative motion in the transverse direction has little change compared with the static contact. Therefore, the stress distribution on the cross section in the rolling state is still symmetrical. However, the action area of longitudinal friction is large, which will lead to the phenomenon that the stress distribution in Figure 3 is at the surface of the rail and shifts towards the back end of the contact area.

**Figure 4:**
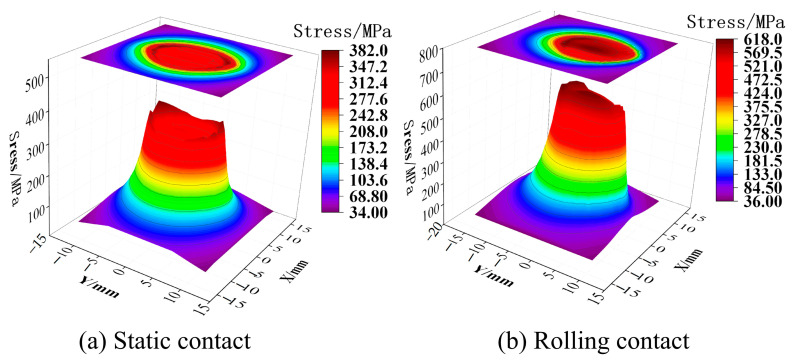
Rail surface stress distribution.

The friction force of the static contact will cause the rail surface stress to become larger in the relative sliding area. As shown in Figure 4a, the stress at the farther position of the contact center produces a ‘bulge‘. The friction of the rolling contact will cause the stress in the contact area to increase compared to the static contact, as shown in Figure 4b. From Figure 2 and Figure 3, it can be seen that whether it is static contact or rolling contact, the stress field of the rail is in the distribution of the cross section and the longitudinal section. The maximum value of the stress under the rolling contact is larger than the maximum value under the static contact, but the difference in the stress level is not very large, ranging within 55 MPa. This is because the maximum value of the stress is in the subsurface, the depth of the friction effect is limited, and the area of the friction effect on the stress is mainly in the rail surface [14]. In Figure 2 and Figure 3, the stress distribution of the cross section and the longitudinal section are in the range near the surface, and the rolling contact is greater than the static contact, which reflects that the friction is mainly in the rail surface area. It can be seen from Figure 4 that under the action of friction, the maximum value of stress at the surface under rolling contact is 240 MPa larger than that of static contact, and the effect of friction is more significant. The stress distribution on the surface under rolling contact is also shifted to the back end of the contact area compared with the static contact. In summary, the maximum value stress of the rail in the two contact states occurs in the subsurface layer. The rolling of the wheel will affect the stress level of the rail surface layer and the longitudinal stress distribution of the surface layer. Therefore, the sampling of the test should include the area from the rail surface layer to the subsurface layer, and the loading method should be along the longitudinal direction of the rail.

## 3. Tests and Methods

### 3.1. Materials and Specimens

**Figure 5:**
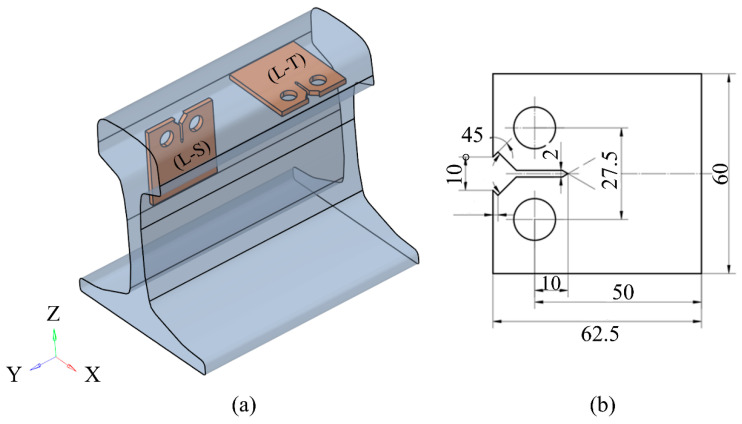
Specimen sampling positions and its dimensions, unit: mm.

The test material is the U71MnG rail steel (China). The compact tension (CT) specimen was designed according to the ASTM-E647 standard. The experimental study was the plane stress state, so the thickness of the specimen was equal to 1 mm. Combined with the simulation results in the second chapter, the sampling method and size of the specimen are shown in Figure 5. During the actual service of the rail steel, the cracks will propagate along the L–T and L–S directions; this study can provide a reference for predicting the residual life of rail steel. The metallographic observation was carried out on the samples along the L–T and L–S directions in Figure 5. The fine scratches and deformations on the surface of the specimens were polished, and the polishing effect reached the mirror. After polishing, the surface of the specimens was immediately corroded with nitric acid with a volume fraction of 4% to prevent the formation of an oxide film on the polished surface from affecting the erosion effect. After the corrosion, the specimens were wiped with alcohol and dried, and the microstructure of the sample surface was observed by an optical microscope.

Figure 5a shows the schematic diagram of the specimen processed along different directions, and Figure 5b shows the geometric size of the specimen. The chemical composition and basic mechanical properties of the materials are shown in Table 1 and Table 2. Before the test, the specimens in different sampling directions were observed and analyzed by the bay Olympus BX51M optical microscope (Tokyo, Japan). The surface of the specimen was polished, the speckle treatment was performed on one side of the specimen surface and the crack propagation was observed and recorded by an industrial camera (Nreeohy, Shenzhen, China) on the other side.

### 3.2. Fatigue Test

**Figure 6:**
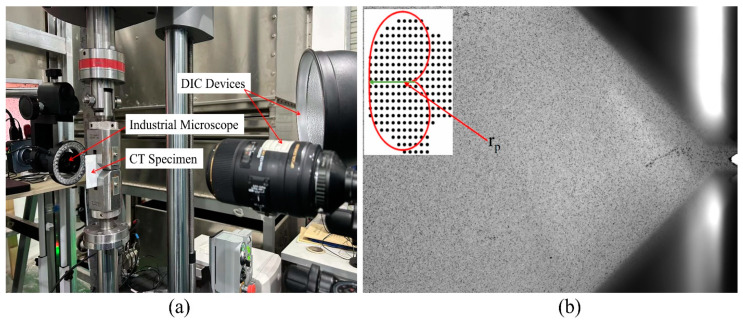
Test loading device, (**a**) DIC acquisition device, (**b**) speckle pattern and the tip radius diagram.

The constant amplitude type I FCG test was carried out on the CT specimen, and the test was carried out on the E10000 ElectroPuls Instron tension–torsion dynamic testing machine (London, UK). Figure 6a shows the field test loading device. The specimen was prefabricated with a length of 2 mm before the test, with the details of the speckle pattern and the tip radius diagram as shown in Figure 6b. The maximum loading *P*_max_ = 1.1 kN. The FCG test was carried out under two stress ratios, *R* = 0.1 and *R* = 0.3. The loading frequency was equal to 20 Hz. All FCG tests were carried out at room temperature. During the test, the industrial camera was used to measure and record the crack length at a specific interval of cycles. At the same time, a digital image correlation (DIC) device, i.e., Revealer 2D-DIC (Luoyang, China), was used to obtain the crack tip displacement field data. Referring to the solution of displacement field parameters, the SIF values of the crack tip are solved by the CJP model. The field of view of DIC was 4096 pixels × 3000 pixels, and the actual size of each pixel was 0.007518 mm.

### 3.3. Method for Calculating ΔK

Considering the influence of various additional stresses caused by the closure effect, the CJP model (biaxial stress) provides the displacement field solution equation [11,12], as shown in Equation (1):

(1) $$\begin{array}{l} 2G\left(u+iv\right)=k\left[-2Bz^{0.5}-2Ez^{0.5}\ln\left(z\right)-\frac{C}{4}z\right]-\\ z\left[-\left(B+2E\right){\overset{-}{z}}^{-0.5}-E{\overset{-}{z}}^{-0.5}\bar{\ln\left(z\right)}-\frac{C}{4}\right]-\\ \left[A{\overset{-}{z}}^{0.5}+D{\overset{-}{z}}^{0.5}\bar{\ln\left(z\right)}-2D{\overset{-}{z}}^{0.5}+\frac{C}{2}\overset{-}{z}\right] \end{array},$$

where *G* is the shear modulus, *u* and *v* are the horizontal and vertical displacement fields, respectively, *κ* = 3 − 4*υ* in the plane strain state, *κ* = (3 − 4*υ*)/(1 + *υ*) in the plane stress state and υ is the Poisson’s ratio. *A*, *B*, *C*, *D* and *E* are the coefficients to be solved, *A* = −*B* = *K*/√(8π), *C* = −*T*, *T* is *T* stress, *D* and *E* are the introduced correction terms, *z* = *r*e^iθ^, *z* is the conjugate complex number of *z* and the effective crack propagation driving factor of the CJP model is:

(2) $$\Delta K_{CJP}=\left(K_{F,\max}-K_{R,\max}\right)-\left(K_{F,\min}-K_{R,\min}\right)$$

(3) $$K_{F}=\underset{r\to 0}{\lim}\sqrt{2\pi r}\left[\sigma_{y}(r,0)+2Er^{-0.5}\ln r\right]=\sqrt{\frac{\pi }{2}}(A-3B-8E)$$

(4) $$K_{R}=\underset{r\to 0}{\lim}\sqrt{2\pi r}\left[\sigma_{x}(r,\pm \pi )\right]=-{\left(2\pi \right)}^{\frac{3}{2}}E$$

(5) $$K_{S}=\underset{r\to 0}{\lim}\sqrt{2\pi r}\left[\sigma_{xy}(r,\pm \pi )\right]=\mp \sqrt{\frac{\pi }{2}}(A+B)$$

where *K*_F,max_, *K*_R,max_ and *K*_F,min_ are *K*_R,min_ are *K*_F_ and *K*_R_ under maximum and minimum stress, respectively.

## 4. Results and Discussion

### 4.1. Microstructure Analysis

**Figure 7:**
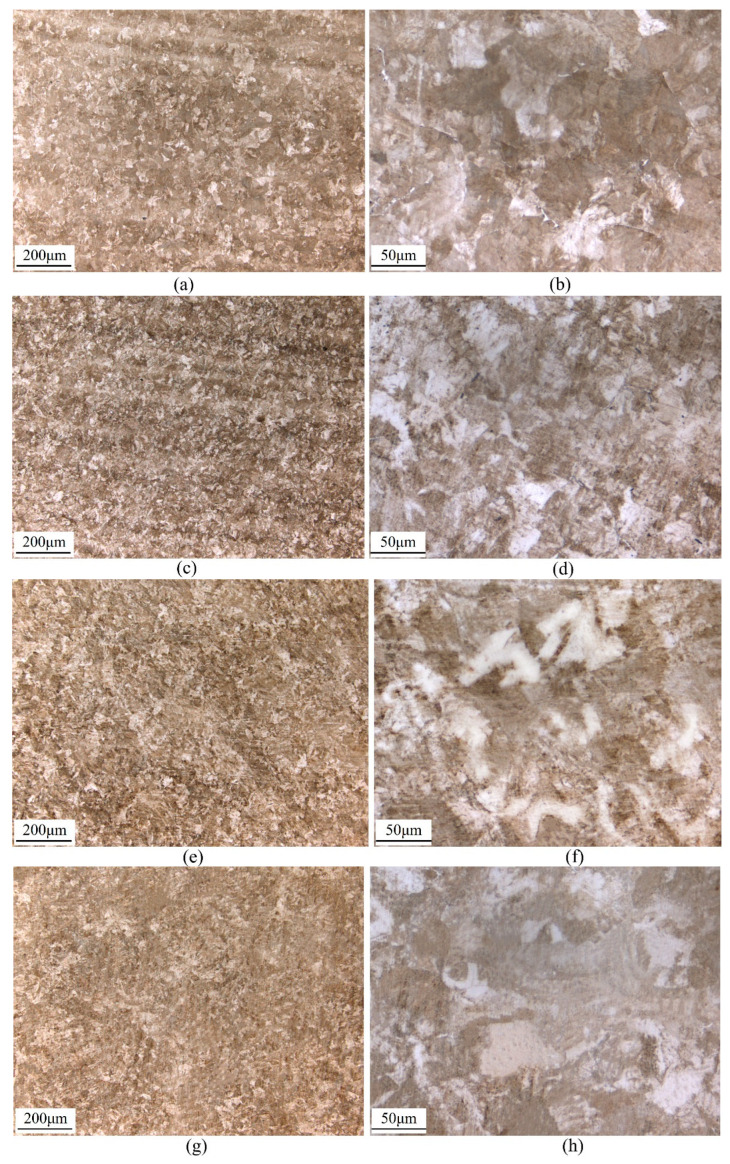
The metallographic structure of U71MnG: (**a**,**b**) in the L–T direction of rail head (CSP), (**c**,**d**) in the L–T direction of rail head (CRP), (**e**,**f**) in the L–S direction of rail head (CSP), (**g**,**h**) in the L–S direction of rail waist (CRP).

In each zone of the rail, the microstructure will be different and will affect the rate of fatigue crack growth. For each of the samples (L–T or L–S), depending on the crack length, the crack will grow through different zones of the rail. The metallographic structure analysis was carried out at the corresponding positions of the crack stable propagation stage (CSP) and the rapid propagation stage (CRP), as shown in Figure 7. L–T is sampled on the parallel surface of the rail head, and the metallographic structure is similar, as shown in Figure 7a–d. L–S samples were taken from the rail head and rail waist. The metallographic structure of the rail head is shown in Figure 7e,f; the rail waist is shown in Figure 7g,h. The results of L–T and L–S microstructure analysis show that the grain size of L–T is smaller than that of L–S. When the microcrack enters another grain from one grain through the grain boundary, the slip direction of the dislocation and the crack propagation direction needs to be changed due to the change of grain orientation. Therefore, the smaller the grain size, the more times the direction needs to be changed in the crack propagation path, and the greater the energy consumption, that is, the higher the toughness of the material [13,14,15].

The metallographic structure of the U71MnG is mainly composed of pearlite and ferrite. The content of ferrite and pearlite in L–T specimens remained at the same level during crack propagation. In the process of crack propagation from the rail head to the rail waist in L–S specimens, the content of ferrite was increased, and the content of pearlite was decreased. Ferrite has good plasticity and toughness but low strength and hardness. Pearlite is a two-phase mechanical mixture of ferrite and cementite. Pearlite has higher strength and hardness than ferrite. The pearlite content in L–T is significantly higher than that in L–S, and the crack propagation resistance of the L–T is higher than that of L–S under the same driving force.

### 4.2. Crack Tip Plastic Zone Characterization Analysis

**Figure 8:**
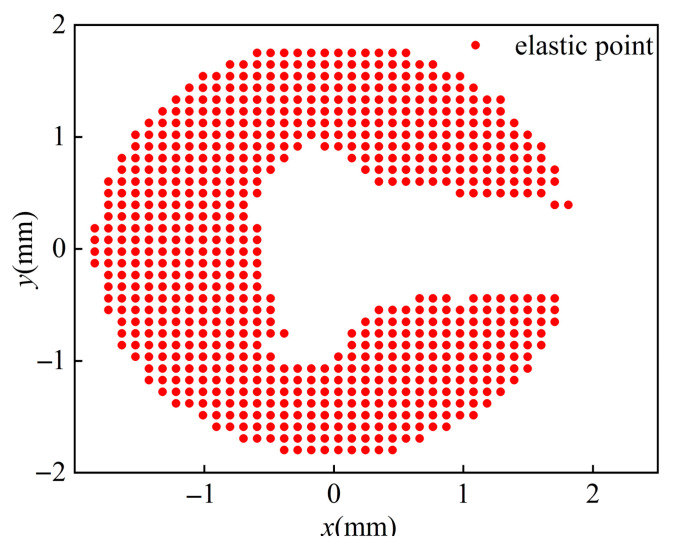
Theoretical plastic zone diagram.

The size and area of the plastic zone at the crack tip have an important influence on the crack growth rate during crack propagation. The CJP model has a good effect on characterizing the shape and closure effect of the plastic zone at the crack tip because the CJP model takes into account the influence of plastic-induced shielding [16,17,18,19]. In this study, the CJP model was used to reveal the effect of closure effect on crack growth rate. In this study, the DIC method is used to obtain the full-field displacement field. In the Matlab software processing solver (Matlab 2021), the ncorr.m program provided by the open-source software (Ncorr V1.2) is used to solve the displacement cloud map and obtain the displacement field data. The strain field is obtained by the full-field strain solution method based on the two-dimensional Savitaky-Golay digital differentiator, and then the stress field is solved by the generalized Hooke’s law, and it is substituted into the Von. Mises yield criterion to obtain the size and shape of the experimental plastic zone. In the process of obtaining the theoretical plastic zone, the coefficient sets *A*, *B*, *C* and *E* of the CJP model need to be obtained. The solution of the coefficient set is very sensitive to the crack tip position and the size of the calculation area. Yang et al. [20] used the L–M nonlinear iterative algorithm to iteratively calculate the crack tip position as an unknown number to avoid the resulting error caused by the inaccuracy of the initial crack tip position. Zhou and Liu et al. [21] derived the relative error between the Westergard model stress component *σ*_y_ and the exact solution stress component σ*_y_ in an infinite plate and proposed that the error is small when the fitting outer diameter is 0.1 times the crack length, and the larger plastic zone can be up to 0.2 times. Thus, more accurate crack tip field parameters can be obtained. As shown in Figure 8, the plastic zone boundary is taken as the inner diameter, the plastic field area is removed, and the fitting outer diameter is selected to be about 0.1 times the crack length to meet the accuracy requirements. The coefficient sets *A*, *B*, *C* and *E* are solved by L–M nonlinear iteration.

**Figure 9:**
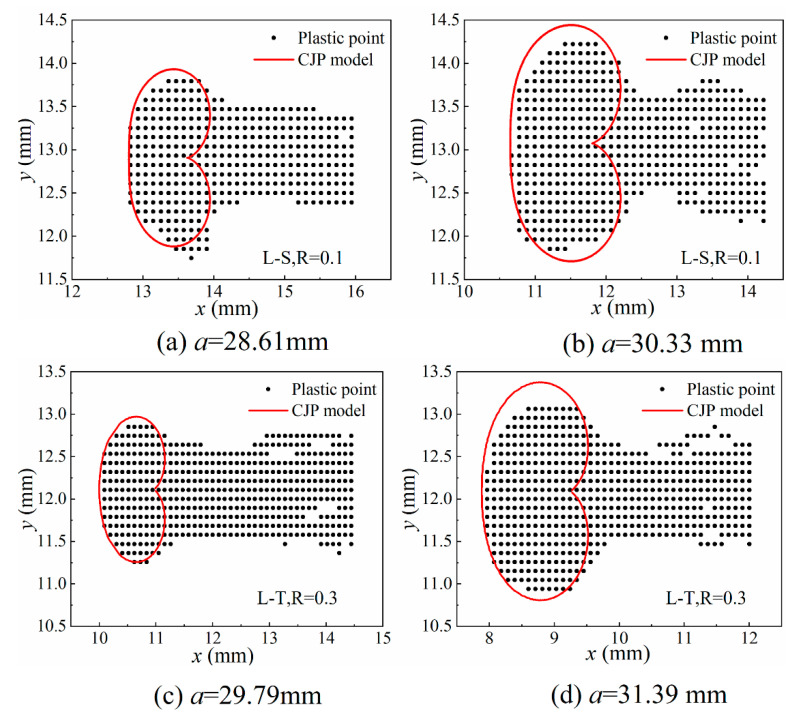
The comparison between the size of the experimental plastic zone and the theoretical plastic zone of the CJP model.

The DIC method has the advantages of a simple optical path, non-contact and full-field measurement, but it has high requirements for image quality. If there are large noise, blur, occlusion and other problems in the image, the accuracy and effect of the algorithm will be affected. The theoretical plastic zone shape can be obtained by using the elastic field data to obtain the CJP model coefficient set, as shown in Figure 9. The black scatter points in the figure are the scatter points of the plastic zone measured experimentally, and the red area is the theoretical plastic zone obtained based on the CJP model. It can be seen from the figure that the size of the plastic zone increases with the increase of the crack length. Comparing the size and shape of the theoretical plastic zone and the experimental plastic zone of the CJP model, the results show that the CJP model can well describe the outline of the experimental plastic zone and has a good description effect in both sampling directions and throughout the crack propagation stage. This provides an accurate basis for using the CJP model to study the crack propagation of U71MnG steel.

### 4.3. Characterization of Crack Tip SIF

**Figure 10:**
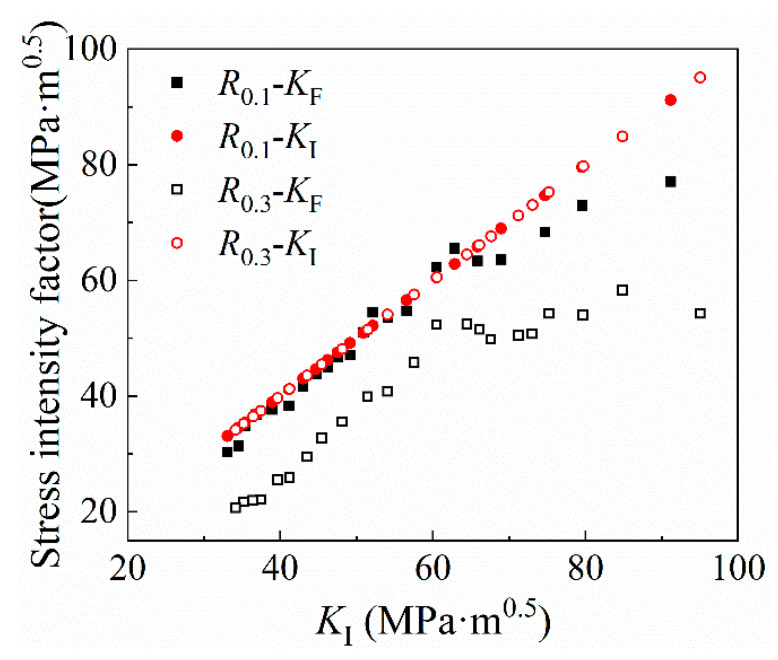
The variation of SIF *K*_F_ with traditional SIF *K*_I_.

**Figure 11:**
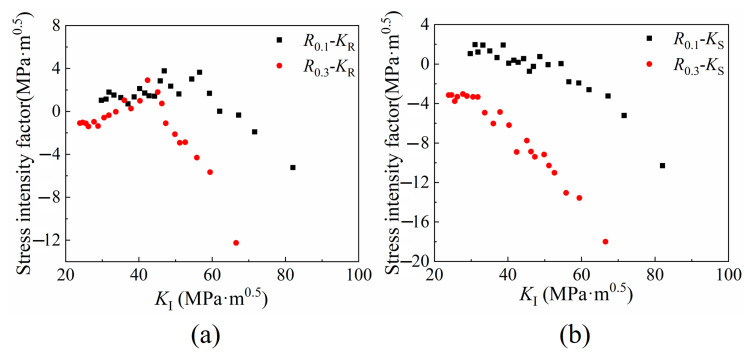
The variation of (**a**) SIF *K*_R_ and (**b**) SIF *K*_S_ with traditional SIF *K*_I_.

In the CJP model, *K*_F_, *K*_R_ and *K*_S_ are the three SIF that characterizes the crack propagation process. *K*_F_ characterizes the open-type SIF that drives crack propagation. *K*_F_ and *K*_I_ are the SIF that drives crack propagation. *K*_I_ is the traditional SIF of type I crack. *K*_R_ is the SIF corresponding to the stress parallel to the crack propagation direction in the elastic zone due to the different Poisson ratios between the elastic material and the plastic material. *K*_S_ is the SIF corresponding to the shear stress along the crack surface caused by the elastic–plastic compatible stress. In Figure 10 and Figure 11, the variation trend of the three SIF with the traditional SIF *K*_I_ is shown. In the initial stage of crack propagation, the values of *K*_F_, *K*_R_ and *K*_S_ remain unchanged, which is because there is no large deformation in the initial stage of crack propagation and the crack propagation speed is relatively small. As the crack propagates, the values of *K*_F_, *K*_R_ and *K*_S_ change uniformly in a certain direction. In Figure 10, the *K*_F_ value corresponding to *R* = 0.3 is lower in the early stage of crack propagation, while the propagation curve at *R* = 0.1 is almost the same as that of traditional *K*_I_. In the later stage of crack propagation, due to the plastic-induced crack closure effect, the *K*_F_ is lower than the traditional SIF. Figure 10 well describes the difference between the crack closure effect and the traditional solution of the crack tip SIF. In Figure 11, *K*_R_ is the SIF that hinders crack propagation. At the beginning of crack propagation, the value changes around 0, and the SIF value at *R* = 0.1 is higher than the SIF value at *R* = 0.3. *K*_S_ and *K*_R_ have similar trends, and both *K*_S_ and *K*_R_ gradually decrease with crack propagation. The *K*_R_ value obtained from this test is different from the previous observation results of polycarbonate, 2024-T3 aluminium alloy, and industrial pure titanium [22,23,24]. Taking industrial pure titanium as an example, the characteristics of industrial pure titanium are not high strength but good plasticity, which is quite different from the U71MnG rail steel used in this study, and the differences in microstructure will have a certain impact on *K*_R_ and *K*_S_ values.

**Figure 12:**
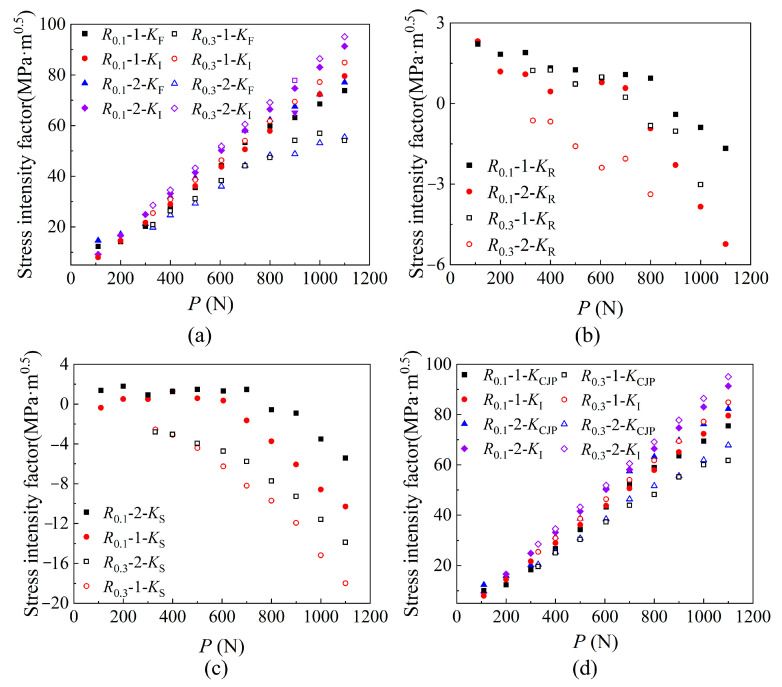
SIF of (**a**) *K*_F_, (**b**) *K*_R_, (**c**) *K*_S_, and (**d**) *K*_CJP_ change with load *P* (In the legend, 1 and 2 represent different crack lengths, respectively. Under the condition of *R* = 0.1, 1 and 2 represent crack lengths of 32.756 mm and 33.940 mm, respectively. Under the condition of *R* = 0.3, 1 and 2 represent crack lengths of 32.037 mm and 33.513 mm, respectively).

The variation trend of *K*_F_, *K*_R_, *K*_S_ and *K*_CJP_ values with loading at two different crack lengths is plotted and compared, as shown in Figure 12. The SIF of *K*_F_, *K*_R_ and *K*_S_ change slowly with the loading at the initial stage of expansion and change sharply when exceeding its certain value, which is correlated with the crack opening force. Because the SIF of the CJP model can better characterize the crack closure phenomenon, it is not necessary to consider the influence of crack opening SIF (*K*_op_). *K*_CJP_ is a SIF considering the driving force and hindrance force of crack propagation. The value is obtained based on the CJP model, which can well characterize the plastic-induced crack closure effect and ensure accuracy [10,15,25,26].

By analyzing the data in Figure 12, the *K*_CJP_ value and the traditional *K*_I_ value show a linear growth trend, and the *K*_CJP_ value obtained by the high-stress ratio solution under the same crack length is smaller. Combined with the analysis of the relationship between the plastic zone area and ∆*K*_CJP_ in Section 4.2, the existence of residual compressive stress in the plastic zone at the crack tip makes the SIF value that drives crack propagation’s need to consume some energy to neutralize the influence of residual compressive stress, which indirectly leads to the decrease of crack propagation rate.

**Figure 13:**
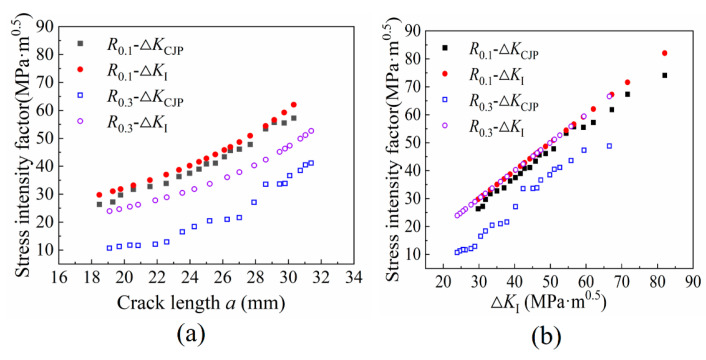
The variation trend of SIF (**a**) Variation trend of *a*-∆*K*_CJP_ (**b**) Variation trend of ∆*K*_I_-∆*K*_CJP_.

Figure 13 shows the variation trend of the SIF ∆*K*_CJP_ with the crack length *a*. The results obtained based on the CJP model are lower than the typical values. This is because there is a plastic effect at the crack tip during the entire crack propagation stage, so ∆*K*_CJP_ is lower than the traditional ∆*K*_I_, which reflects the elastic-plastic-induced shielding force of the CJP model. The calculated Δ*K*_CJP_ under a high-stress ratio is smaller than that under a low-stress ratio and is quite different from the traditional Δ*K*_I_.

### 4.4. Crack Growth Rate

**Figure 14:**
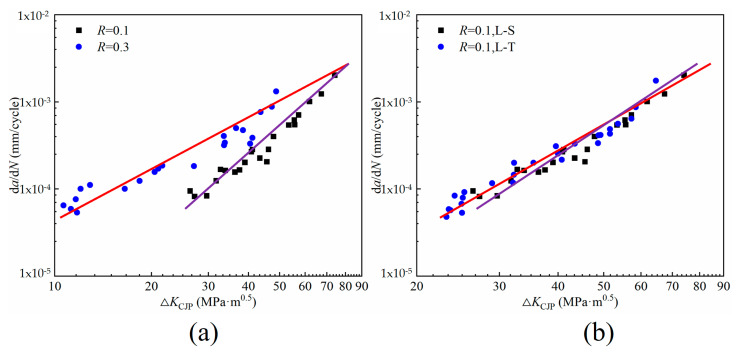
Data of FCG rate. (**a**) The crack growth rate of specimens selected from the L–T direction under different stress ratios, (**b**) the crack growth rate of specimens at different orientation under the same stress ratio.

This study explores the influence of different specimen directions and different stress ratios on the crack growth rate of rail steel materials, as shown in Figure 14a shows the crack growth rate curves under two stress ratios. The results show that the crack growth rate is greatly affected by the stress ratio. At high-stress ratios, the crack growth rate curve with *R* = 0.3 is shifted to the left compared with *R* = 0.1. The results of Ostashin et al. are consistent with the results of this paper, which believed that the cyclic crack growth resistance of steel decreases for high loading amplitudes as the load ratio increases [27]. In addition, Ostashin et al also study different parameters used for the construction of the diagrams of fatigue crack-growth rate, and the crack-tip opening displacement and local strain energy are more sensitive to the structural and mechanical characteristics of the materials than the ordinary d*a*/d*N*−Δ*K* diagrams [28]. In Figure 14b, the crack propagation rate of the specimen along the L-S direction is slightly higher than that in the L-T direction under the same stress ratio. There are differences in the grain size of the metallographic structure sampled in different directions. Under the same crack propagation distance, the energy consumed by the crack to expand to the vicinity of the grain boundary in the specimen with a smaller grain size is higher than that in the sample with a larger grain size. Therefore, the crack propagation rate in Figure 14b is different, which also leads to different cycles in the crack propagation process.

The data in the analysis chart can also be found that the crack growth rate curve shows good normalization and a good linear effect. It is worth mentioning that the driving parameter Δ*K*_CJP_ expressed by the CJP model does not need to be corrected. This is because the CJP model takes into account the influence of plastic-induced shielding, which can directly reflect the displacement state during crack propagation, avoiding the deviation of the traditional SIF range from the real results under high plasticity. In the previous work [29,30], the CJP model effectively reflects the distribution of experimental data and has a good fitting effect. Compared with the traditional parameter Δ*K*, Δ*K*_CJP_ is directly solved by the crack tip displacement field and can be used to describe the fatigue crack growth rate of materials without correction.

## 5. Conclusions

Based on the results of finite element simulation, combined with the DIC method and microstructure analysis, this study describes the crack propagation in the U71MnG rail steel under different stress ratios and different orientations of specimens based on the CJP model. The results are as follows:

1. The maximum value of stress of the wheel–rail static contact and rolling contact is in the subsurface of the rail, the stress level of the rail in the rolling contact state is higher than that in the static contact state and the stress distribution of the longitudinal surface layer of the rail changes under the influence of wheel rolling, which lays a foundation for the sampling and loading method of the rail fatigue test and verifies the rationality of the test.
2. The grain size in the L–T direction is smaller than that in the L–S direction. The finer the grain, the more times the direction needs to be changed in the crack propagation path, the greater the energy consumption and the lower the crack propagation rate.
3. The CJP model can well describe the contour of the experimental plastic zone. The CJP model can well characterize the influence of the crack tip compatible stress and crack closure effect on crack propagation under different stress ratios.
4. The crack growth rate curve under the high-stress ratio moves to the left relative to the low-stress ratio, and the difference in grain size leads to the similarities and differences in crack growth rate.

## Figures and Tables

**Table 1:** Mass fraction of chemical composition of U71MnG rail steel.

C	Si	Mn	P	S	Cr	V	Al
0.65–0.76	0.15–0.58	0.70–1.20	<0.03	<0.025	-	-	<0.01

**Table 2:** U71MnG rail steel mechanical properties parameter table.

Elastic Modulus	Ultimate Tensile Strength	Yield Strength	Elongation	Poisson Ratio
*E*/MPa	*σ*_b_/MPa	*σ*_s_/MPa	*δ* (%)	𝜐
210,000	≥880	780	≥10	0.28

## Data Availability

The raw/processed data required to reproduce these findings cannot be shared at this time as the data also forms part of an ongoing study.
